# Enhancing the Thermostability of *Serratia plymuthica* Sucrose Isomerase Using B-Factor-Directed Mutagenesis

**DOI:** 10.1371/journal.pone.0149208

**Published:** 2016-02-17

**Authors:** Xuguo Duan, Sheng Cheng, Yixin Ai, Jing Wu

**Affiliations:** 1 State Key Laboratory of Food Science and Technology, Jiangnan University, Wuxi, Jiangsu Province, China; 2 School of Biotechnology and Key Laboratory of Industrial Biotechnology Ministry of Education, Jiangnan University, Wuxi, Jiangsu Province, China; 3 Department of Biology, South University of Science and Technology of China, Shenzhen, Guangdong Province, China; University Paris South, FRANCE

## Abstract

The sucrose isomerase of *Serratia plymuthica* AS9 (AS9 PalI) was expressed in *Escherichia coli* BL21(DE3) and characterized. The half-life of AS9 PalI was 20 min at 45°C, indicating that it was unstable. In order to improve its thermostability, six amino acid residues with higher B-factors were selected as targets for site-directed mutagenesis, and six mutants (E175N, K576D, K174D, G176D, S575D and N577K) were designed using the RosettaDesign server. The E175N and K576D mutants exhibited improved thermostability in preliminary experiments, so the double mutant E175N/K576D was constructed. These three mutants (E175N, K576D, E175N/K576D) were characterized in detail. The results indicate that the three mutants exhibit a slightly increased optimal temperature (35°C), compared with that of the wild-type enzyme (30°C). The mutants also share an identical pH optimum of 6.0, which is similar to that of the wild-type enzyme. The half-lives of the E175N, K576D and E175N/K576D mutants were 2.30, 1.78 and 7.65 times greater than that of the wild-type enzyme at 45°C, respectively. Kinetic studies showed that the *K*_m_ values for the E175N, K576D and E175N/K576D mutants decreased by 6.6%, 2.0% and 11.0%, respectively, and their *k*_cat_/*K*_m_ values increased by 38.2%, 4.2% and 19.4%, respectively, compared with those of the wild-type enzyme. After optimizing the conditions for isomaltulose production at 45°C, we found that the E175N, K576D and E175N/K576D mutants displayed slightly improved isomaltulose yields, compared with the wild-type enzyme. Therefore, the mutants produced in this study would be more suitable for industrial biosynthesis of isomaltulose.

## Introduction

Isomaltulose (commonly referred to as palatinose) is a structural isomer of sucrose, and its physical and organoleptic properties are similar to those of sucrose [1, 2]. However, unlike sucrose, isomaltulose is a non-cariogenic, nutritional sugar with low-caloric content, great acid stability, and low hygroscopicity [3]. Isomaltulose is digested more slowly than sucrose, so it decreases the rate of monosaccharide release into the blood. This slows the rate of insulin release, promoting its use for diabetics and nondiabetics. Isomaltulose is currently used as an industrial precursor to isomaltitol, which is prepared from isomaltulose by catalytic hydrogenation [4].

Sucrose isomerase (EC 5.4.99.11), also known as isomaltulose synthase, has been purified and characterized from various bacterial species, including *Serratia plymuthica* ATCC 15928 [5], *Protaminobacter rubrum* CBS 547.77 [6], *Klebsiella* sp. LX3 [7], *Klebsiella pneumoniae* NK33-98-9[8], *Pantoea dispersa* UQ68J [9], *Enterobacter* sp. FMB-1 [10], *Erwinia rhapontici* NX-5 [4, 11, 12], *Pseudomonas mesoacidophila* MX-45 [13]. A series of the corresponding genes have been cloned and designated *pal*I [5, 7, 8, 10, 11], *smu*A [6], *sim* [9] and *mut*B [13]. During the isomerization of sucrose to isomaltulose and trehalulose, small amounts of glucose and fructose are produced as by-products [5, 7, 9, 14]. The ratio of isomaltulose to trehalulose varies depending on the bacterial strain from which the enzyme was obtained and the reaction conditions [5, 8–11, 15, 16]. Some purified sucrose isomerases produce predominantly isomaltulose (75–85%) [9, 11, 16, 17], whereas others produce mainly trehalulose (90%) [15]. X-ray crystal structural information has been reported for the sucrose isomerase (SmuA) from *P*. *rubrum* CBS 547.77 [6], the isomaltulose synthase (PalI) from *Klebsiella* sp. LX3 and *E*. *rhapontici* NX-5 [12, 16], and the trehalulose synthase (MutB) from *P*. *mesoacidophila* MX-45 [18].

The temperature optima of most bacterial sucrose isomerases are between 30°C and 40°C [5, 6, 8–11, 13]. The half-life of *Klebsiella* sp. LX3 sucrose isomerase is only 1.8 min at 50°C [7, 11]. The sucrose isomerase from *Enterobacter sp*. FMB-1 retained more than 70% of its activity after storage at 50°C for 2 h, but its activity was drastically reduced at 60°C [11]. The sucrose isomerase from *K*. *pneumoniae* NK33-98-8 lost all of its activity after incubation at 50°C for 40 min [8]. Due to the thermolability of these sucrose isomerases, the enzymatic production of isomaltulose is usually carried out at temperatures ranging from 30°C to 40°C [19]. However, the sucrose solution that serves as the substrate is vulnerable to microbial contamination at these temperatures. So, enhancing the thermostability of sucrose isomerase will improve the performance of large-scale isomaltulose production using this enzyme.

Protein engineering is a powerful strategy to improve the stability of a protein. Protein engineering can be classified into three general categories: rational design, a semi-rational approach, and irrational design (directed evolution). One of the rational design methods, B-factor-based site-directed mutagenesis [20], has been used to enhance the thermostability of *Bacillus subtilis* lipase [21], *Candida antarctica* lipseB (CalB) [22], *Rhizomucor miehei* lipase (RML) [23] and *Coprinus cinereus* peroxidase (CiP) [24]. Generally, B-factor-based mutation consists of two steps. First, the appropriate sites at which rigidity needs to be increased are chosen on the basis of atomic displacement parameters obtained from X-ray structural data, namely the B-factor (or B-value). Then, the selected sites are either substituted with other amino acids predicted to be more rigid through computational modeling (RosettaDesign), or they are subjected to saturation mutagenesis. B-factor-based site selection combined with saturation mutagenesis has been used to develop thermostable RML [23], while B-factor-based site selection combined with RosettaDesign was employed to develop thermostable CalB [25].

In this study, the sucrose isomerase from *S*. *plymuthica* AS9 was expressed in *Escherichia coli* BL21(DE3), and the recombinant enzyme produced was characterized. Because the wild-type enzyme was not stable at high temperature, its sequence was modified using site-directed mutagenesis based on reported B-factors and RosettaDesign-based selection of alternate amino acids. The resulting mutants displayed enhanced thermostability while retaining enzymatic activity.

## Materials and Methods

### Bacterial strains, vectors and materials

The gene encoding sucrose isomerase (NCBI accession number YP_004505648.1) from *S*. *plymuthica* AS9 was optimized based on the preferred codon usage of *E*. *coli* and synthesized by Shanghai Generay Biotech Co. Ltd. (Shanghai, China). This synthetic gene was incorporated into the modified secretion-expression vector pET-24a-*ompA*, which was constructed using pET-24a(+) (Novagen; Madison, WI, USA) and *ompA* as backbone and secretion signal peptide coding sequence respectively, to generate the expression vector pET-24a-*ompA*/*palI* (S1 and S2 Files). *E*. *coli* JM109 (TakaRa, Dalian, China) was used as host for gene cloning, while *E*. *coli* BL21(DE3) (Novagen) was used for sucrose isomerase expression. The restriction enzymes, T_4_ DNA ligase, *Dpn* I, agarose gel DNA purification kit, pMD18-T simple vector and *E*. *coli* JM109 were purchased from TaKaRa (Dalian, China). Isomaltulose and trehalulose were purchased from Sigma (Shanghai, China). All other chemicals were obtained from Sinopharm Chemical Reagent Co. Ltd. (Shanghai, China).

### Media

Luria-Bertani (LB) medium that contained 10.0 g L^-1^ tryptone, 5.0 g L^-1^ yeast extract and 10.0 g L^-1^ NaCl was used as seed cultures. Shake-flask cultures were carried out in a modified TB medium that contained 12.0 g L^-1^ tryptone, 24.0 g L^-1^ yeast extract, 16.4 g L^-1^ K_2_HPO_4_, 2.3 g L^-1^ KH_2_PO_4_, 5.0 g L^-1^ glycerol.

### Design and construction of mutants

Considering that AS9 PalI and SmuA differ in only one amino acid, structure of the wild-type sucrose isomerases was obtained by single point mutation through pymol using the crystal structure of SmuA from *P*. *rubrum* CBS 547.77 (PDB ID, 3GBD). Homology models of structures of the mutant sucrose isomerases were constructed based on the crystal structure of SmuA (PDB ID, 3GBD), using the EMBL-EBI server. The B-factor profile of 3GBD was obtained from the RCSB Protein Data Bank. Six amino acids having high B-factors were selected for mutagenesis. The selection of more rigid amino acids was performed using the RosettaDesign server (http://rosettadesign.med.unc.edu). In which, any selected amino acid was substituted by the other 19 amino acids, and the total energy (△G) for the wild type protein and the mutants was calculated, respectively. The mutant with the lowest total energy in silicon was obtained by site-directed mutagenesis using the wild type protein as parent. ClustalX was used to construct multiple sequence alignments, and then Boxshade server was used for sequence coloring (http://www.ch.embnet.org/software/BOX_form.html). Site-directed mutagenesis was performed using a PCR method with the plasmid pET-24a-*ompA*/*pal*I as the template. The sequences of the forward and reverse primers containing the appropriate base changes are listed in Table 1. The PCR products were digested with *Dpn* I and then used to transform *E*. *coli* JM109. Successful mutation of the original plasmid was confirm by DNA sequencing. The recombinant plasmids designed for the expression of wild-type and mutant enzymes were used to transform the chemically competent *E*. *coli* BL21(DE3) cells.

**Table 1:** Primers used for site-directed mutagenesis.

Primers	Sequences (5’ to 3’ direction)^a^
K174D-For	TTTTGGAAGGACGCA**GAT**GAGGGCCAAGCGCCG
K174D-Rev	CGGCGCTTGGCCCTC**ATC**TGCGTCCTTCCAAAA
E175N-For	TGGAAGGACGCAAAA**AAT**GGCCAAGCGCCGAAT
E175N-Rev	ATTCGGCGCTTGGCC**ATT**TTTTGCGTCCTTCCA
G176D-For	AAGGACGCAAAAGAG**GAT**CAAGCGCCGAATAAC
G176D-Rev	GTTATTCGGCGCTTG**ATC**CTCTTTTGCGTCCTT
S575D-For	ATTATTGACAGCAAT**GAT**AAAAACGTGGTGAAA
S575D-Rev	TTTCACCACGTTTTT**ATC**ATTGCTGTCAATAAT
K576D-For	ATTGACAGCAATAGC**GAT**AACGTGGTGAAAAAG
K576D-Rev	CTTTTTCACCACGTT**ATC**GCTATTGCTGTCAAT
N577K-For	CAGCAATAGCAAA**AAA**GTGGTGAAAAAGAAT
N577K-Rev	ATTCTTTTTCACCAC**TTT**TTTGCTATTGCTG

^a^ Bold bases represent the coding sequence of the mutated amino acid.

### Expression and purification of wild-type sucrose isomerase and mutants

To express the enzymes, *E*. *coli* BL21(DE3) harboring the constructed plasmids were inoculated into 50 mL LB medium containing 30 μg/mL kanamycin in a 250 mL flask and grown at 37°C in a rotary shaker at 200 rpm. The overnight seed culture was then diluted (1:25) into 50 mL of modified TB medium supplemented with kanamycin (30 μg/mL) and glycine (7.5 g/L) at 37°C, with shaking at 200 rpm, until the absorbance reached 1.5. Protein expression was induced with the addition of isopropyl â-D-thiogalactopyranoside (IPTG) to a final concentration of 0.2 mM. Protein expression was then allowed to proceed at 25°C for 36 h.

The culture supernatant were collected by centrifugation at 4000 g and 4°C for 20 min [26, 27], and then solid ammonium sulfate was slowly added to the culture supernatant, with gentle agitation, to a final concentration of 60% (w/v). The precipitated protein was obtained by centrifugation at 10000 g at 4°C for 20 min and dissolved in buffer A (50 mM citric acid/sodium phosphate buffer, pH 5.3), and then dialyzed against buffer A overnight. The sample was then filtered (0.22 μm) and subjected to cation-exchange chromatography using a Fast Protein Liquid Chromatography system (AKTA avant; GE Healthcare) equipped with Sepharose high-performance cation-exchange column (160 mm 10 mm, Pharmacia Biotech), pre-equilibrated with buffer A. The column was eluted with a 5 column-volume linear gradient of 0 to 1 M NaCl in buffer A at a flow rate of 1 mL/min. Fractions containing sucrose isomerase activity were pooled. The pooled fractions were aliquoted and stored at -20°C until use.

### SDS-PAGE and protein assay

SDS-PAGE was performed under reducing conditions using a 5% stacking gel and a 12% separating gel. Protein bands were visualized by staining with Comassie Brilliant Blue R-250 dye according to standard procedures. Protein concentration was determined by the Bradford method with bovine serum albumin as the standard.

### Sucrose isomerase activity assay

The activity of sucrose isomerase was measured at 30°C in 50 mM citric acid/sodium phosphate buffer (pH 6.0) containing sucrose at a final concentration of 292 mM. Specifically, 100 μL diluted enzyme solution was mixed with 900 μL of an assay solution and incubated for 15 min. The reaction was stopped by the boiling the mixture in a 100°C water bath for 5 minutes. Afterwards, the sucrose, isomaltulose, trehalulose, glucose, and fructose contents of the assay mixture were quantified using high-performance liquid chromatography system equipped with a refractive index detector, as described in section “HPLC analysis”, below. One unit of sucrose isomerase activity was defined as the amount of enzyme that produced 1 μmol of isomaltulose or trehalulose per min under the conditions described above.

### Effects of pH and temperature on enzyme activity

To determine the optimal pH for enzyme activity, assays were carried out in 50 mM buffers containing citric acid/sodium phosphate (pH 4.0 to 6.0), sodium phosphate (pH 6.0 to 8.0), or glycine-NaOH (pH 8.0 to 9.0). The reaction mixture contained 100 μl dilute purified enzyme (about 1.0–2.0 μg enzyme protein) and 900 μL of 292 mM sucrose, and reactions were incubated at 30°C for 15 min. The optimal temperature was determined between 20°C and 50°C in 50 mM citric acid/sodium phosphate buffer (pH 6.0), using 292 mM sucrose as the substrate. At each temperature, the enzyme was equilibrated in reaction buffer at pH 6.0 for 15 min before addition of the substrate. Reactions were allowed to proceed for 15 min.

To test enzyme stability, the purified enzyme was diluted in 50 mM citric acid/sodium phosphate buffer (pH 6.0) at a protein concentration of 0.25 mg/mL, and then incubated at 40°C or 45°C. Aliquots were removed at different incubation times, and the residual activity was measured at 30°C in the same buffer using 292 mM sucrose as substrate. The initial activity before incubation at 40°C or 45°C was taken as 100%. All enzymatic assays were performed in triplicate, and the results are expressed as the mean ± standard deviation.

### Measurement of Kinetic Parameters

To determine the kinetic parameters, sucrose isomerase was measured by incubating the purified enzyme with different sucrose concentrations (14.6, 29.2, 58.4, 102, 146, 190, 234, 292, 438 and 584 mM) under standard assay conditions. The *V*_max_ and *K*_m_ values were determined by using the nonlinear regression function in GraphPad Prism (GraphPad Software Inc., San Diego, CA). All kinetic parameters presented in this study were determined in duplicate, and the results are presented as the average of these two measurements.

### HPLC analysis

The quantities of sucrose, isomaltulose, trehalulose, glucose and fructose were measured using a D-2000 Elite HPLC system (Hitachi High-Technologies Corporation, Japan) equipped with a Syncronis Amino Column (4.6 mm×250 mm, Thermo scientific, USA) and a refractive index detector (RID). Samples (10 μL) were diluted 5- or 10-fold prior to injection. The mobile phase was acetonitrile-water (80:20), the flow rate was 0.8 mL/min, and the column and detector temperatures were 30°C. The product concentrations were calculated from their respective peak areas by comparing these peak areas with those of standard sugar samples.

## Results

### Sequence analysis and expression of *S*. *plymuthica* AS9 sucrose isomerase in *E*. *coli*

A gene from *S*. *plymuthica* AS9 (NCBI accession number YP_004505648.1) annotated in the NCBI database as encoding an oligo-1,6-glucosidase was screened in this study. A protein sequence analysis of this gene revealed that it encodes 600 amino acids with a calculated molecular mass of 67.56 kDa, and the isoelectric point was 6.86. The first 25 amino acids of the N-terminal end of the protein (Met^1^ to Ala^25^) was predicted by the SignalP 4.1 Server [28] to comprise a signal peptide. As shown in S3 File, the protein sequence exhibited 71%, 72%, 73%, 81%, 97.67%, and 99.83% identity with sucrose isomerases of *Enterobacter* sp. FMB-1 (ACF42098.1), *Klebsiella* sp. LX3 (AAK82938.1), *K*. *pneumoniae* NK33-98-8 (AAM96902.1), *E*. *rhaponitici* NX-5 (ADJ56407.2), *S*. *plymuthica* ATCC15928 (CAF32990.1) and *P*. *rubrum* CBS574.77 (CAF32988.1), respectively [6, 8–11, 16]. All seven of these enzymes have a short, conserved motif (RLDRD) that is uniquely conserved in sucrose isomerases but not in oligo-1,6-glucosidases. This short RLDRD sequence may control the product specificity of sucrose isomerase and is important in the isomerization process [29]. Therefore, it seemed that the “oligo-1,6-glucosidase” from *S*. *plymuthica* AS9 was more likely be a sucrose isomerase than an oligo-1,6-glucosidase.

**Fig 1:**
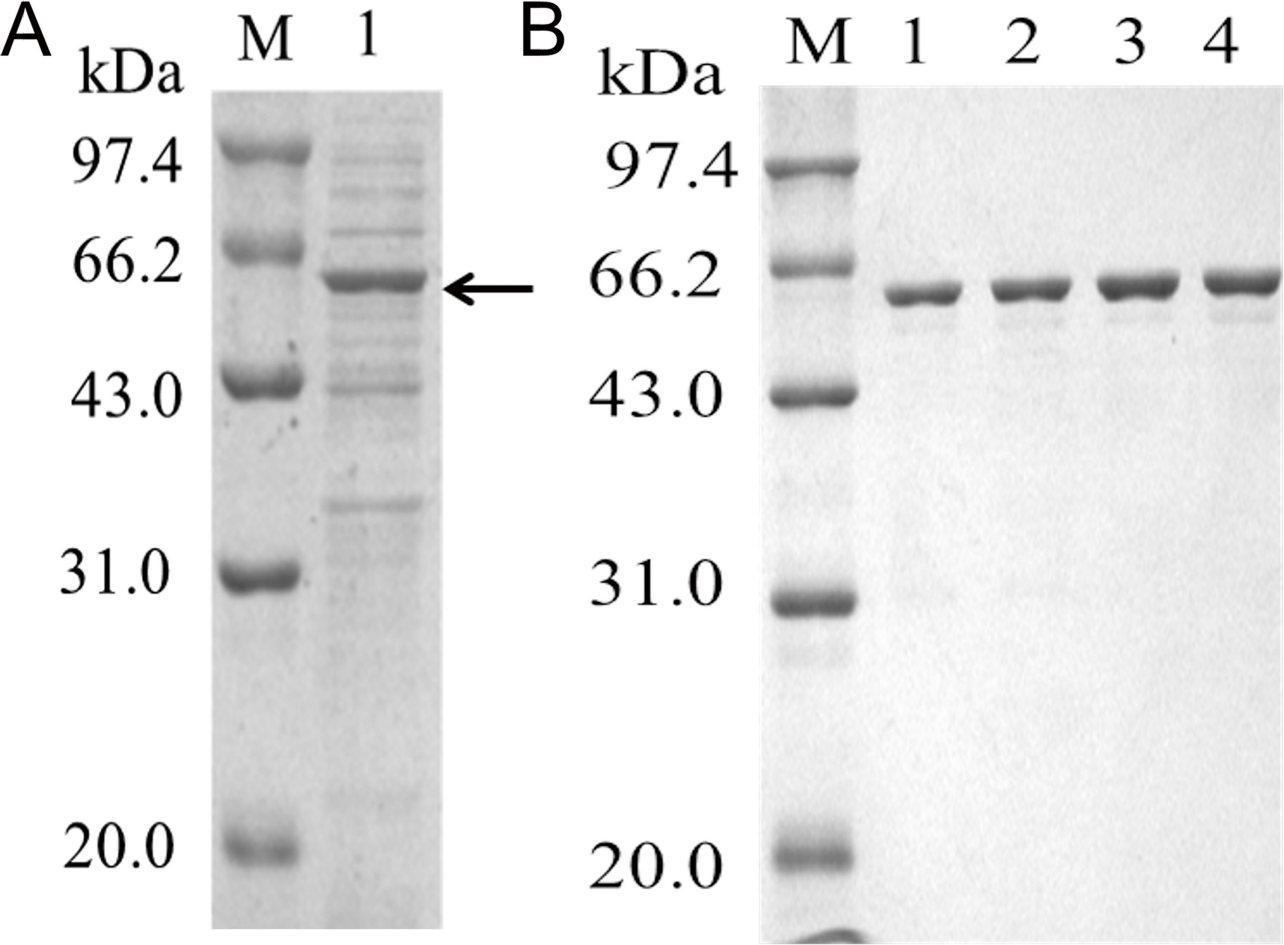
SDS-PAGE analysis of recombinant *S*. *plymuthica* AS9 PalI and its variants expressed in *E*. *coli* BL21(DE3). (A) *Lane* M, molecular weight markers; *Lane* 1, culture supernatant of the wild-type enzyme; Sucrose isomerase is indicated by the black arrow. (B) *Lane* M, molecular weight markers; *Lane* 1, purified wild-type enzyme; *Lane* 2, purified E175N; *Lane* 3, purified K576D; *Lane* 3, purified E175N/K576D.

To test this hypothesis, the recombinant expression and analysis of the protein was undertaken. A codon-optimized synthetic *pal*I gene prepared and inserted into the expression vector pET-24a-*ompA*, which was then used to transformed into chemically competent *E*. *coli* BL21(DE3). SDS-PAGE analysis of the protein expressed by this system showed one major band of protein with a molecular mass of approximately 65 kDa (Fig 1A). Activity assays showed that the ration of enzyme in the supernatant and periplasmic space was 69.7% and 30.3%, respectively. The recombinant enzyme was purified by ammonium sulfate fraction and high-performance cation-exchange chromatography. The purified protein was determined to be homogeneous by SDS–PAGE (Fig 1B). Its activity was measured in citric acid/sodium phosphate buffer using sucrose as the substrate. The results showed that the sucrose was isomerized to isomaltulose and trehalulose, and the enzyme did not display oligo-1,6-glucosidase activity. The specific activity of the purified enzyme was 957.5 U/mg. Thus, the recombinant enzyme is, in fact, a sucrose isomerase.

### Selection of the mutagenesis site and construction of sucrose isomerase variants

**Fig 2:**
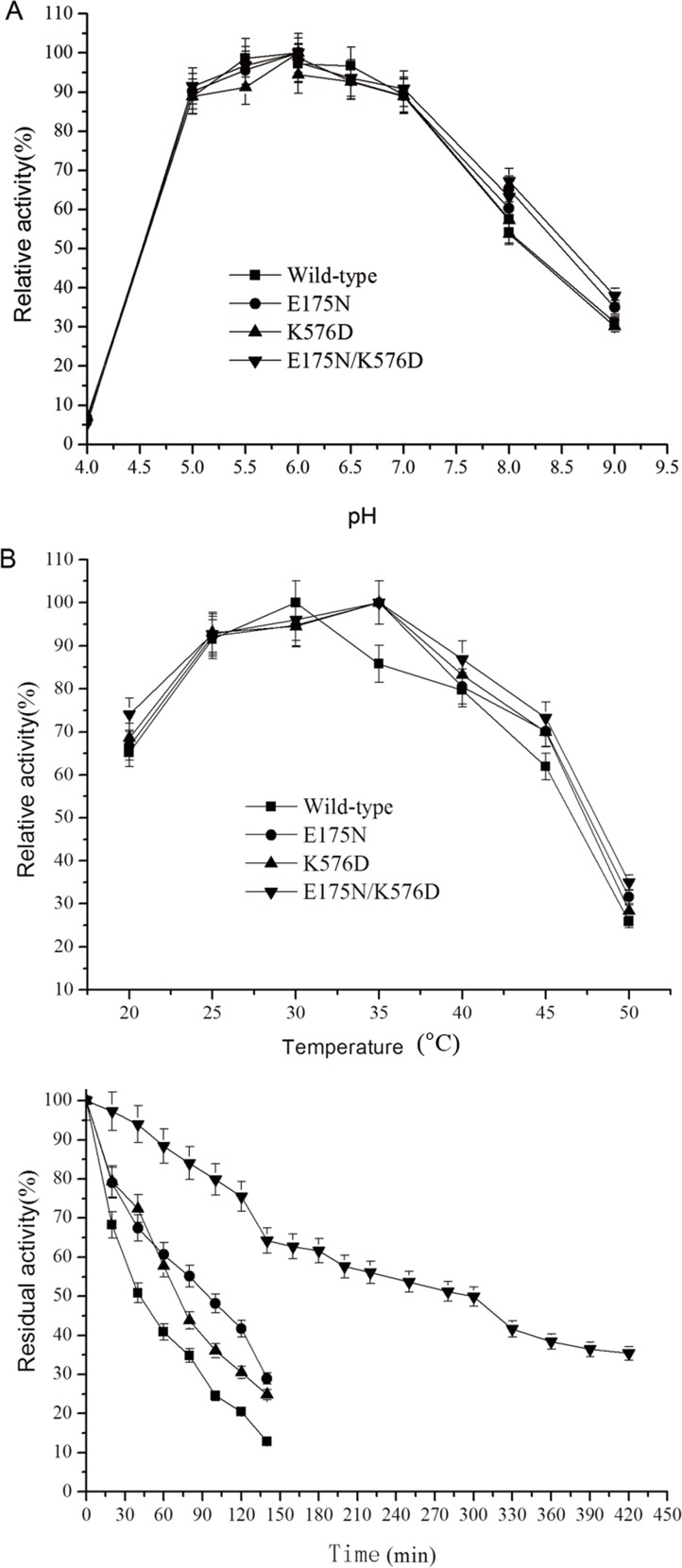
Effects of pH and temperature on the enzyme activities and stabilities of recombinant *S*. *plymuthica* AS9 PalI and its variants. (A) Optimal pH values of recombinant *S*. *plymuthica* AS9 PalI and its variants. The assays were carried out at 30°C with sucrose as substrate in buffers of various pH. (B) Optimal temperatures of recombinant *S*. *plymuthica* AS9 PalI and its variants. The enzymes were incubated with sucrose at temperatures from 20°C to 50°C in 50 mM citrate-phosphate buffer (pH 6.0). (C) Thermostabilities of recombinant *S*. *plymuthica* AS9 PalI and its variants. The enzymes were incubated at 45°C and pH 6.0 for the indicated periods of time. The activity without heat treatment was defined as 100%. Error bars correspond to the standard deviations of three independent determinations. The figure was generated using Origin 9.0.

Characterization of the recombinant enzyme, which we will refer to as As9 PalI, showed that the optimal pH and optimal temperature were 6.0 and 30°C (Fig 2A and 2B), respectively. The half-life of AS9 PalI at pH 6.0 and 45°C was only 39.2 min, and the wild-type enzyme tended to inactive when used in the production of isomaltulose. In order to improve the enzyme for industrial applications, site directed mutagenesis based on B-factor analysis was used to improve the thermostability of the enzyme.

**Fig 3:**
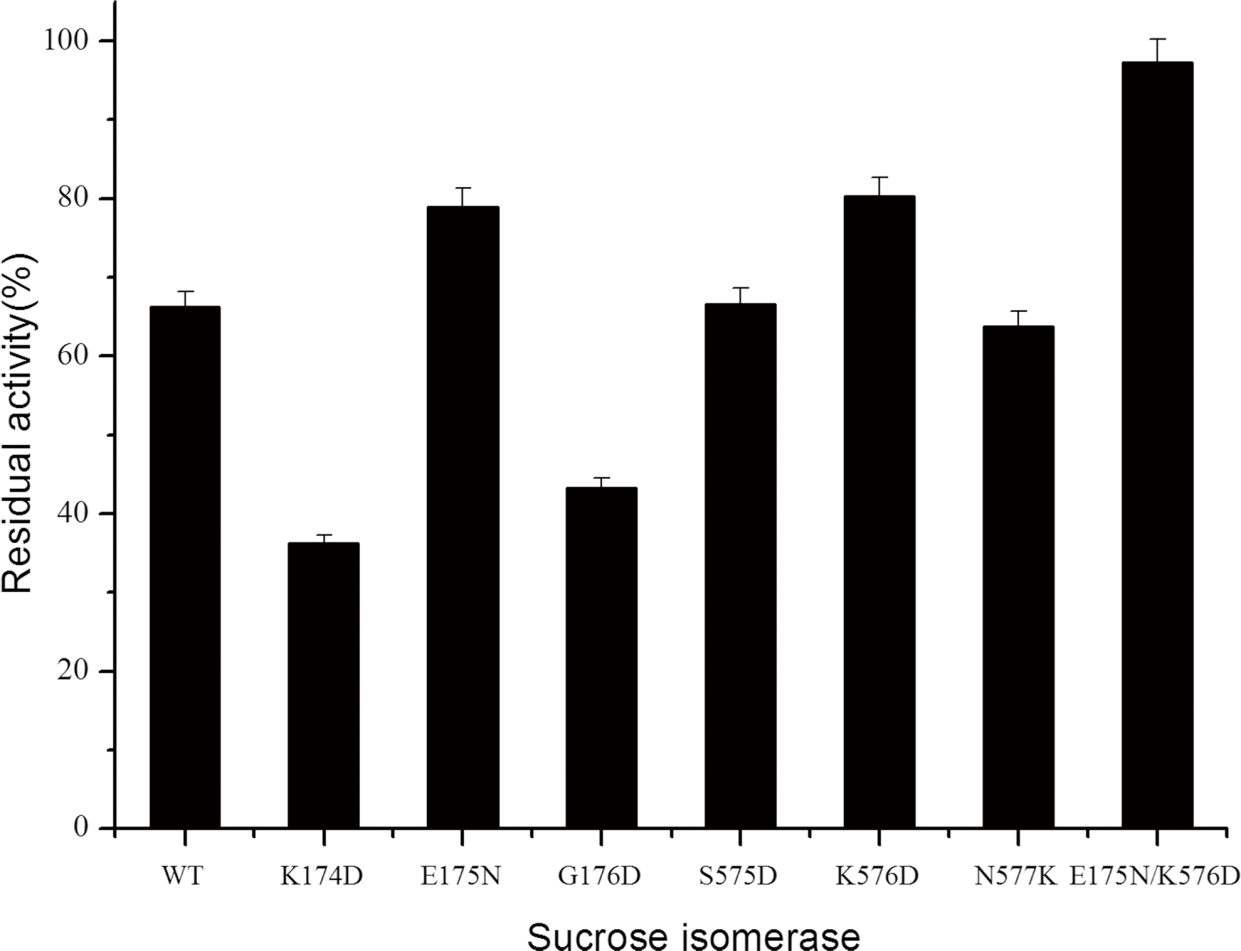
The residual activity of recombinant *S*. *plymuthica* AS9 PalI and its variants after incubation at 45°C and pH 6 for 20 min. The activity of recombinant enzyme without heat treatment was defined as 100%. The figure was generated using Origin 9.0.

A model of the three-dimensional structure of AS9 PalI was constructed based on the X-ray crystal structure of the SmuA from *P*. *rubrum* CBS 574.77 (PDB: 3GBD) using the EMBL-EBI server. Considering that AS9 PalI and SmuA differ in only one amino acid, the homology model predicts that they have almost identical three-dimensional structures and B-factor parameters. Based on this analysis, six AS9 PalI residues (Table 2) with higher B factors were chosen as sites for mutagenesis. To determine the most appropriate substitutions, amino acids expected to be more rigid than the original amino acids were selected using RosettaDesign [30]. Six point mutants of AS9 PalI (K576D, N577K, S575D, K174D, E175N and G176D) were constructed, and their thermostabilities at 45°C and pH 6.0 were evaluated using a 20-min incubation. As shown in Fig 3, wild-type AS9 PalI retained 66.2% of its original activity, whereas the E175N, K576D, K174D, G176D, S575D and N577K mutants retained 79.0%, 80.3%, 36.2%, 43.3%, 66.6% and 63.8% of their individual original activity, respectively. These results demonstrate that point mutants E175N and K576D exhibit better thermostability than the wild-type enzyme. To assess the potential for interaction between these mutations, the double mutant E175N/K57D was constructed and characterized. E175N/K576D retained 97.3% of its original activity when incubated at 45°C, pH 6.0 for 20 min (Fig 3).

**Table 2:** The residues with higher B-factor.

Residue	B-factor
Lys576	44.58
Asn577	42.49
Ser575	42.08
Lys174	37.03
Glu175	34.25
Gly176	33.89

These three mutant enzymes were the purified to apparent homogeneity. As shown in Fig 2B, the purified recombinant mutant enzymes displayed a molecular mass of approximately 65 kDa, which is similar to that of the wild-type enzyme. Mutants E175N, K576D and E175N/K576D displayed increases in specific activity of 6.3%, 9.2% and 27.3%, respectively, compared with that of the wild-type enzyme (Table 3).

**Table 3:** Kinetic parameters for wild-type enzyme and mutants using sucrose as substrate.

Enzyme	Specific activity (U·mg^-1^)	*K*_m_ (mM)	*k*_cat_ (s^-1^)	*k*_cat/_*K*_m_ (s^-1^· mM^-1^)
**Wild-type**	957.5 ± 47.8	30.1 ± 1.5	992.8 ± 49.6	33.0±1.65
**E175N**	1017.6 ± 50.9	28.1 ± 1.4	1280.3 ± 64.0	45.6 ±2.28
**K576D**	1045.7 ± 52.3	29.5 ± 1.8	1014.3 ± 50.7	34.4 ± 1.72
**E175N/K576D**	1218.9 ± 60.9	26.8 ±1.3	1055.8 ± 52.8	39.4 ± 1.97

### Effects of pH and temperature on the activity of the wild-type enzyme and its variants

The optimal pHs of these enzymes were determined by assaying them at various pH values (4.0 to 9.0). The optimal pH value for all three of the mutant enzymes was 6.0, which was similar to that of the wild-type enzyme. All the enzymes retained >88% relative activity over a wide pH range (5.0 to 7.0) (Fig 2A). The optimal temperatures of these enzymes were measured in the range of 20°C to 50°C, using sucrose as substrate in 50 mM citrate-phosphate buffer (pH 6.0). While the optimal temperature of the wild-type enzyme was 30°C, mutants E175N, K576D and E175N/K576D displayed an optimal temperature of 35°C (Fig 2B). At 40°C, wild-type AS9 PalI retained 79.7% of its maximum activity, whereas mutants E175N, K576D and E175N/K576D retained 80.5%, 83.2%, and 86.8% of their maximum activities, respectively.

To evaluate the thermostabilities of the enzymes, they were incubated at 45°C, pH 6.0, and their residual activities were assayed after different incubation times. As shown in Fig 2C, the thermostabilities of the mutants were noticably greater than that of the wild-type enzyme, with E175N/K576D displaying the greatest increase in thermostability. At 45°C and pH 6.0, the half-life of wild-type enzyme was 39.2 minutes. Whereas, the half-lives of E175N, K576D and E175N/K576D were 90.2 minutes, 69.8 minutes and 300 minutes, respectively, which are 2.30, 1.78 and 7.65 times greater than that of the wild-type enzyme.

### Kinetic parameters of wild-type enzyme and its variants

The kinetic parameters of wild-type enzyme and its variants were measured at 30°C. As shown in Table 3, compared to that of the wild-type AS9 PalI, the *K*_m_ values of E175N, K567D and E175N/K576D decreased by 6.6%, 2.0% and 11.0%, respectively. This means that the mutagenesis increased the substrate affinity. Furthermore, the results showed that the *k*_cat_ of E175N, K576D and E175N/K576D mutants were 1.3-, 1.0- and 1.1- fold that of wild-type enzyme, respectively. Thus, all three mutants display enhanced catalytic constants. Moreover, the catalytic efficiencies (*k*_cat_/*K*_m_ values) of E175N, K576D and E175N/K576D were increased by 38.2%, 4.2% and 19.4%, respectively.

### Isomaltulose production by the wild-type enzyme and its variants

**Fig 4:**
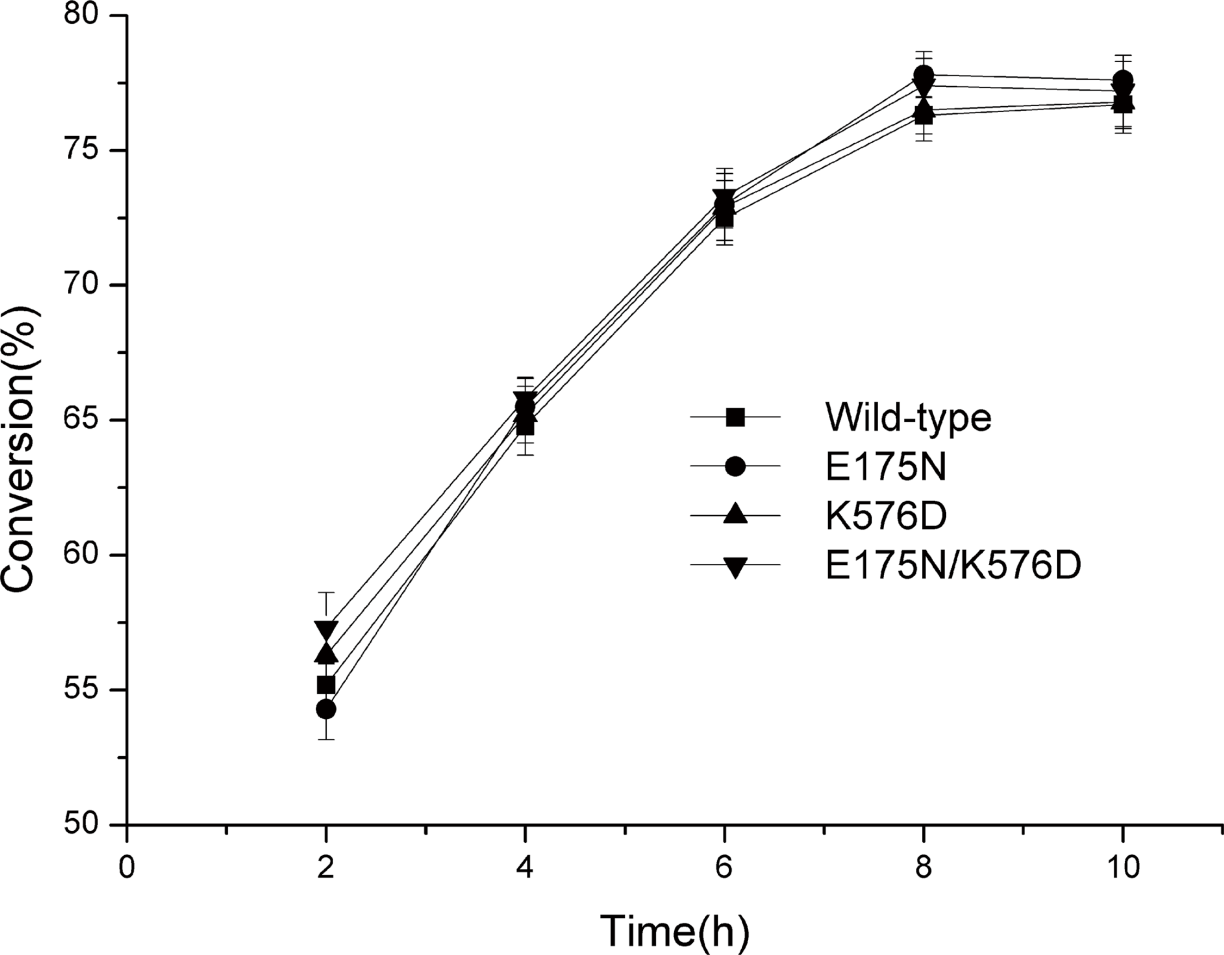
Isomaltulose production by the recombinant *S*. *plymuthica* AS9 PalI and its variants. Sucrose (400 g/L) was incubated with 40 U sucrose isomerase per gram sucrose at 45°C and pH 6.5. The quantities of isomaltulose produced were determined by HPLC. Error bars correspond to the standard deviations of three independent determinations. The figure was generated using Origin 9.0.

The reaction conditions for isomaltulose production by the wild-type enzyme and its three mutants were optimized in this study. The maximal yields of isomaltulose with all of the enzymes were obtained with 400 g/L sucrose, pH 6.5, 45°C, 40 U sucrose isomerase per gram sucrose, and 8 h of incubation. Thus, the wild-type and mutant enzymes share the same optimal reaction conditions. After 8 h of incubation, the maximal yields of isomaltulose reached 76.3%, 77.8%, 76.8% and 78.4% for the wild-type enzyme, E175N, K576D and E175N/K576D, respectively (Fig 4). Thus, under optimal conditions, mutants E175N, K576D and E175N/K576D produced 2.0%, 1.0% and 2.8% more isomaltulose than the wild-type, respectively. This indicates that the mutagenesis not only improved the thermostability of AS9 PalI, it also had small beneficial impacts on the production of isomaltulose using this sucrose isomerase.

## Discussion

**Fig 5:**
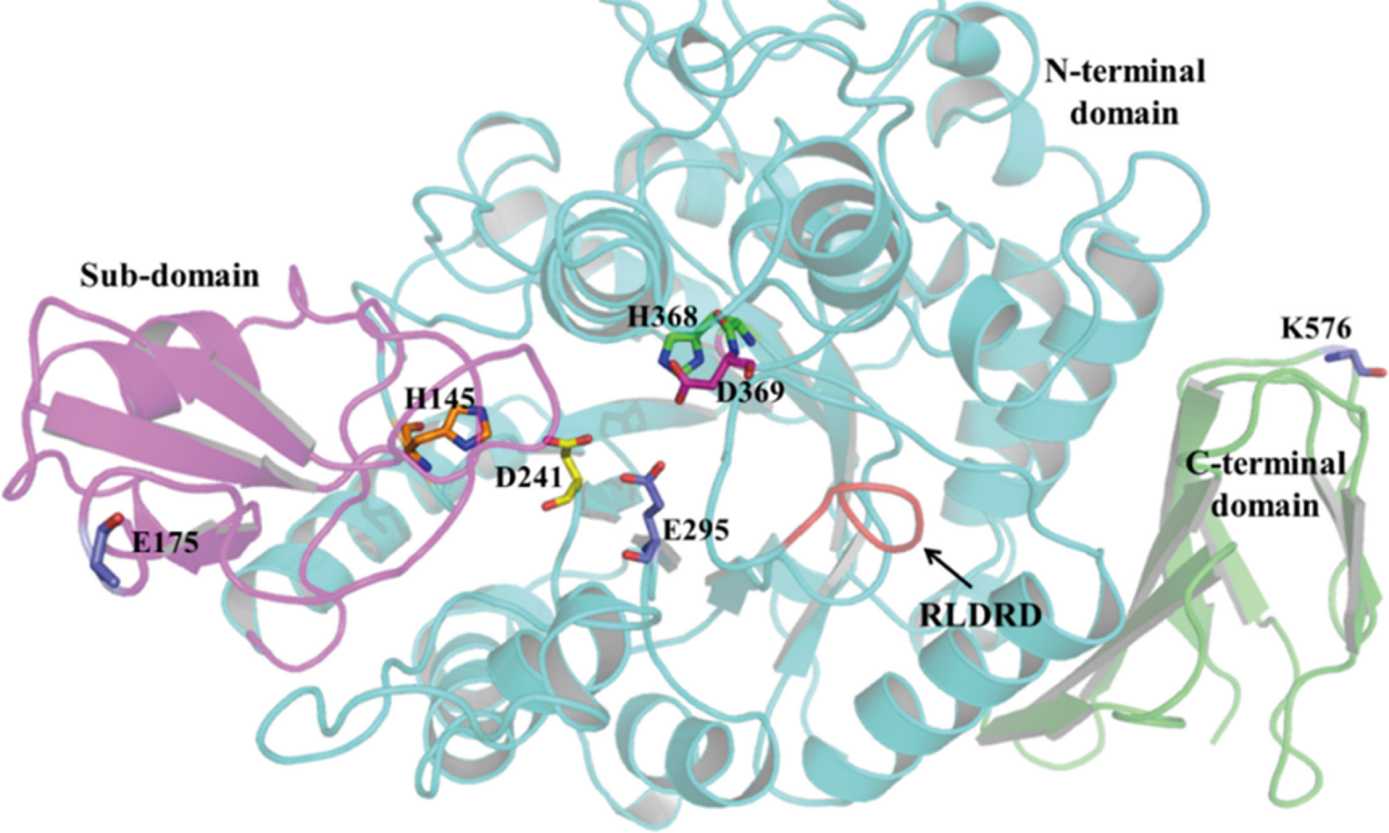
Homology model of the structure of recombinant *S*. *plymuthica* AS9 PalI based on the X-ray crystal structure of the sucrose isomerase from *P*. *rubrum* (PDB:3GBD). The catalytic pocket contains five conserved residues (Asp^241^, Glu^295^, Asp^369^, His^145^ and His^368^) that participate in substrate binding and hydrolysis. These residues are rendered with sticks.The unique, short RLDRD motif of the isomerization region of *S*. *plymuthica* AS9 PalI is rendered in red and indicated by the black arrow. The N-terminal catalytic (β/α)_8_-barrel is rendered in green, the sub-domain in magenta, and the C-terminal domain in cyan. Residues Glu175 and Lys576, rendered with sticks, are positioned from the catalytic center and isomerization region. The figure was generated using PyMol.

In this study, a sequence alignment showed that the *S*. *plymuthica* AS9 PalI, which was annotated as an oligo-1,6-glucosidase in NCBI, exhibited high sequence identity with the sucrose isomerases from *P*. *dispersa* UQ68J, *Enterobacter* sp. FMB-1, *Klebsiella* sp. LX3, *K*. *pneumoniae* NK33-98-8, *E*. *rhaponitici* NX-5, *S*. *plymuthica* ATCC15928 and *P*. *rubrum* CBS574.77 [5–11]. A unique, conserved, short motif (RLDRD), which has been demonstrated to be responsible for sucrose isomerization [16], was found in AS9 PalI. The sequence alignment also showed that AS9 PalI contains a potential catalytic triad (Asp^241^, Glu^295^, and Asp^369^) and two histidine residues (His^145^ and His^368^) that are highly conserved in α-amylase family enzymes. As shown in Fig 5, the model structure of AS9 PalI comprises three domains: an N-terminal catalytic domain, a sub-domain, and the C-terminal domain [16]. These characteristics suggested that AS9 PalI is a member of glycoside hydrolase family 13. Furthermore, the gene encoding *S*. *plymuthica* AS9 PalI was heterogenously expressed in *E*. *coli*, and the sucrose isomerase activity of the resulting protein was assayed. The results demonstrated that *S*. *plymuthica* AS9 PalI is a sucrose isomerase.

The recombinant AS9 PalI was purified and characterized. It showed an optimal temperature of 30°C, which is similar to those of most sucrose isomerases [5, 7–9, 11]. Moreover, it showed that the thermostability of the wild-type AS9 PalI was low. It retained 66.2% and 40.0% of its original activity after 20 min at 45°C and after incubation at 50°C for 8 min, respectively. The enzyme was inactive when incubated at the 50°C for approximately 15 min.

In the present study, in order to improve the thermostability of AS9 PalI, B-factor-based site-directed mutagenesis was applied to enhance the stability of the enzyme. The AS9 PalI was shown to have a three-dimensional structure almost identical to that of the sucrose isomerase from *P*. *rubrum* CBS 574.77, with which it differs in primary structure by a single amino acid. Therefore, six residues with higher B-factors were chosen based on the B-factor information of SmuA. Six AS9 PalI mutants (K576D, N577K, S575D, K174D, E175N and G176D) were designed by using RosettaDesign, and the thermostabilites of the mutants were evaluated.

Based on above analysis, six point mutants were constructed. The mutants E175N and K576D, which showed enhanced thermostability were selected, and the double mutant E175N/K576D was constructed for further study. Our results showed that the half-lives of E175N, K576D and E175N/K576D was 2.30-fold, 1.78-fold, 7.65-fold higher than that of the wild-type, respectively, at 45°C. Compared with the wild-type enzyme, all mutants displayed a shift in optimal temperature from 30 to 35°C. These results demonstrate that all of the mutants display improved thermostability, compared with the wild-type enzyme. Although sucrose isomerase and oligo-1,6-glucosidase exhibit significant differences in catalytic function, they display very similar structures. The oligo-1,6-glucosidases from the *Geobacillus caldoxylosilyticus* (accession no. P29094) displays good thermostability [31]. Therefore, an amino acid sequence alignment of the two enzymes was constructed. The sequence aliment of AS9 PalI and oligo-1,6-glucosidase showed that Lys576 and Glu175 in AS9 PalI were clearly different from the aspartic acid and asparagine residues present in the oligo-1,6-glucosidase. It is interesting that RosettaDesign, when predicting more stable mutants based upon those residues with higher B-factors recommended that residues E175 and K576 be changed to E175N and K576D.

**Fig 6:**
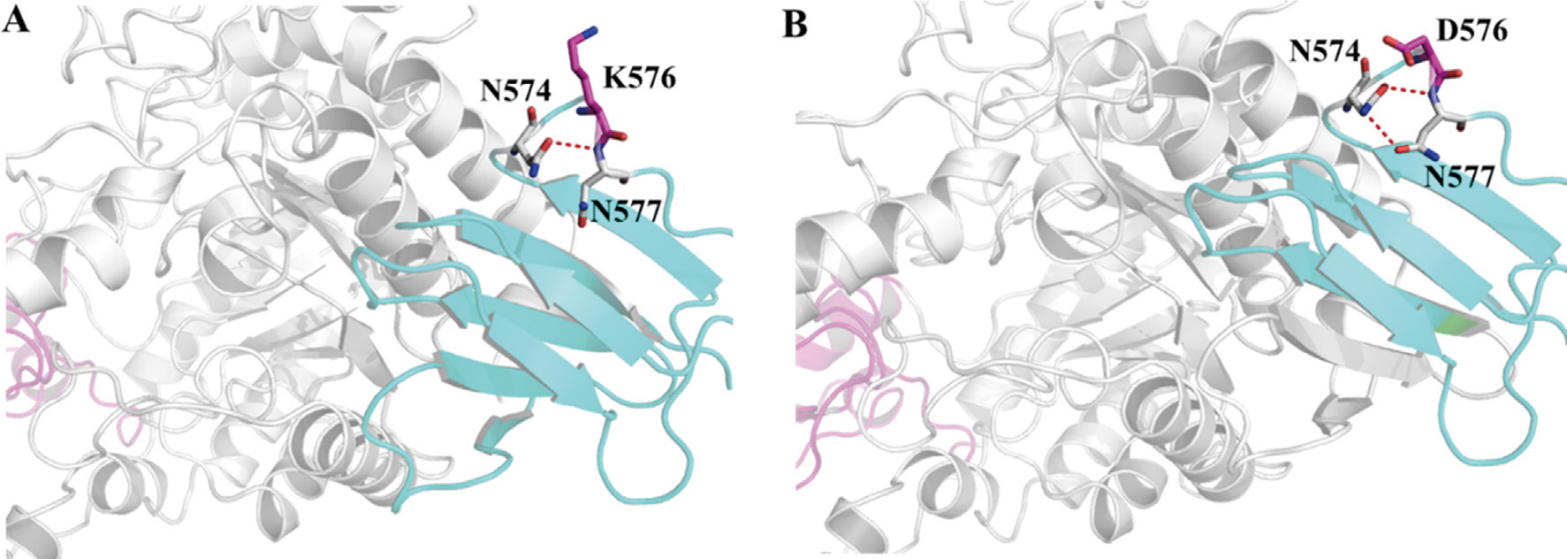
Local views of the structural changes introduced by mutation of *S*. *plymuthica* AS9 PalI at position 576. (A) The wild-type enzyme, showing primarily the N- and C-terminal domains; (B) The K576D mutant, showing primarily the N- and C-terminal domains. The C-terminal domains are rendered in cyan. The N-domains are rendered in white. Hydrogen bonds are indicated as red stippled lines. The residues at position 576 are rendered with sticks. This figure was generated using PyMol.

Improvements in the thermostability of proteins are affected by many factors, including increasing hydrophobic interactions, loop stabilization, and reduction of the entropy of unfolding [32]. Previous strategies of introducing hydrogen bonds, salt bridges or disulfide bridges into a protein have successfully improved the thermal stability of pullulanase [33], cyclodextrin glycosyltransferase [34] and lipase B [24]. Further analysis shows that some hydrogen bonds were introduced into AS9 PalI to enhance the thermostablity of enzyme. As shown in Fig 5, Lys576 and Glu175 are located in the C-terminal domain and the sub-domain, respectively. The B-factor of Lys576 is the highest and the B-factor of Glu175 is relatively high, indicating higher flexibility of these two amino acid residues, which may decrease the stability of the protein. Decreased flexibility may increase the stability of the protein. In this study, Lys576 and Glu175 were converted to Asp576 and Asn175, which were predicted to be more rigid by computational modeling using RosettaDesign. Further analysis showed that mutation of Lys576 to glutamic acid was predicted to form a new hydrogen bond, which formed between Asn574 and Asn577 in the C-terminal domain (Fig 6). It is well known that hydrogen bonding plays important role in the thermal stability of proteins. This new formed hydrogen bonding tends to confer reduced flexibility of the C-terminal loop and thus enhancing the thermal stability of the enzyme. In addition to these single mutations, the double mutant E175N/K576D not only introduced a new hydrogen bond into the C-terminal domain, but also induced subtle alterations to the structure of the whole protein, which may have made the structure more compact. The RosettaDesign server uses Monte Carlo optimization with simulated annealing to search for amino acids that pack well on the target structure, bury their hydrophobic atoms and satisfy hydrogen bonding potential of polar atoms. RosettaDesign has been experimentally validated, it has been used to create a protein with a novel structure [35, 36], and stabilize naturally occurring proteins [37, 38, 39].

Our results showed that the mutations not only influenced the thermostability of AS9 PalI, they also increased the substrate affinity and catalytic efficiency. The residues Lys576 and Glu175 are far from the catalytic center and isomerization region. However, these mutations may have caused a portion of the structure outside the catalytic center and isomerization region to become more compact, which produced a positive effect on the kinetic parameters.

## Conclusion

In the present study, the sucrose isomerase (PalI) of *S*. *plymutic* AS9 was expressed in *E*. *coli* BL21(DE3), purified and characterized. Mutation of Glu175 to asparagine and Lys576 to aspartic acid led to improvements in the thermostability and catalytic efficiency of sucrose isomerase. Notably, the E175N and E175N/K576D mutants showed the highest catalytic efficiency and thermostability, respectively. These enhanced properties indicate that these mutants may be more suitable choices for industrial applications. Significantly, our work confirms that B-factor-based site-directed mutagenesis is an efficient strategy to improve the thermostability of an enzyme. This is the first report of improvements in the thermostability and catalytic efficiency of pullulanase achieved by site-directed mutagenesis.

## Supporting Information

S1 File Plasmid profile of expression vector pET-24a-OmpA/palI. (PDF)

S2 File DNA and Amino acid sequence. (PDF)

S3 File Amino acid sequence alignments of glycoside hydrolase family 13. The crucial conservation residues (Asp241, Glu295, and Asp369, His145 and His368) are indicated by hollow square. The unique short motif “RLDRD” of isomerization region in sucrose isomerase are indicated by red hollow rectangular.
(PDF)
