# Supplementary figures and images for: Enhancing the Thermostability of Serratia plymuthica Sucrose Isomerase Using B-Factor-Directed Mutagenesis
Source: PLoS One. 2016 Feb 17;11(2):e0149208. doi: 10.1371/journal.pone.0149208 (PMC4757035; doi:10.1371/journal.pone.0149208)

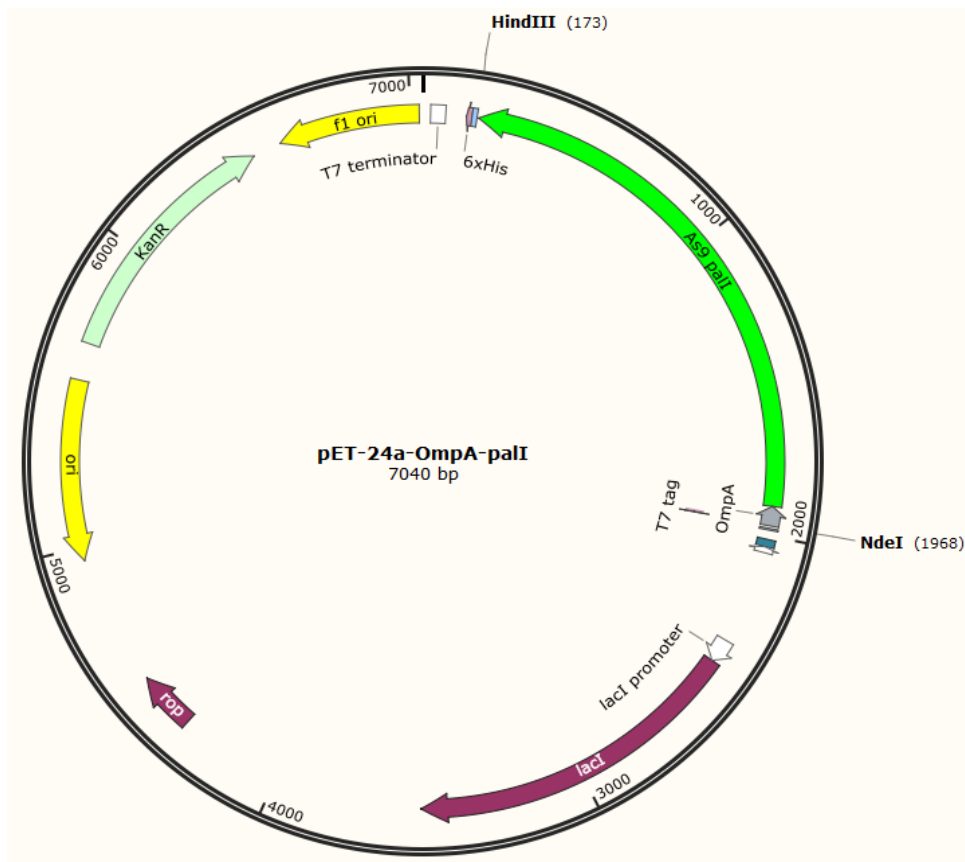

**Fig. S1. Plasmid profile of expression vector pET-24a-OmpA-palI**

Supplement: S1 File — (PDF) [file pone.0149208.s001.pdf]
