# Supplementary material for: Enhancing the Thermostability of Serratia plymuthica Sucrose Isomerase Using B-Factor-Directed Mutagenesis
Source: PLoS One. 2016 Feb 17;11(2):e0149208. doi: 10.1371/journal.pone.0149208 (PMC4757035; doi:10.1371/journal.pone.0149208)
Supplement: S2 File — (PDF) [file pone.0149208.s002.pdf]

DNA sequence of *Omp*A/*pal*I

```
1      ATGAAAAAGA CAGCTATCGC GATTGCAGTG GCACTGGCTG GTTTCGCTAC CGTAGCGCAG
61     GCCTTTGGCA CGCAACAGCC GCTGCTGAAC GAGAAAAGCA TCGAGCAAAG CAAGACCATT
121    CCGAAGTGGT GGAAAGAGGC GGTTTTCTAT CAGGTGTATC CGCGCAGCTT TAAGGACACC
181    AATGGCGATG GTATCGGCGA CATCAACGGC ATTATCGAGA AACTGGACTA CCTGAAGGCG
241    CTGGGCATCG ACGCGATTTG GATCAATCCG CACTACGACA GCCCAAATAC CGATAACGGT
301    TACGACATTC GTGACTATCG TAAGATTATG AAAGAATACG GTACGATGGA GGATTTCGAC
361    CGCCTGATTA GCGAGATGAA GAAACGTAAT ATGCGTCTGA TGATTGACGT TGTTATCAAC
421    CATACCAGCG ACCAGAATGA ATGGTTCGTG AAGAGCAAGA GCAGCAAAGA TAATCCATAT
481    CGTGGCTACT ACTTTTGGAA GGACGCAAAA GAGGGCCAAG CGCCGAATAA CTATCCGAGC
541    TTCTTTGGTG GTAGCGCGTG GCAAAAGGAT GAGAAGACCA ATCAATATTA CCTGCATTAC
601    TTTGCGAAAC AACAACCGGA CCTGAATTGG GATAACCCAA AGGTGCGTCA AGATCTGTAT
661    ACCATGCTGC GTTTCTGGCT GGATAAAGGC GTGAGCGGCC TGCGCTTTGA TACCGTTGCG
721    ACCTACAGCA AAATCCCAGA TTTCCCGAAC CTGACCCAAC AGCAACTGAA AAACTTTGCC
781    GCCGAGTATA CGAAAGGTCC GAACATTCAC CGTTACGTGA ATGAAATGAA CAAGGAGGTG
841    CTGAGCCACT ATGATATCGC GACCGCGGGT GAGATTTTTG GTGTGCCGCT GGATCAGAGC
901    ATTAAGTTCT TTGATCGTCG TCGTGATGAA CTGAATATCG CATTTACCTT TGACCTGATC
961    CGTCTGGATC GTGACAGCGA TCAGCGTTGG CGTCGTAAGG ATTGGAAACT GAGCCAGTTC
1021   CGTCAGATCA TCGACAATGT TGATCGCACC GCCGGTGAGT ACGGTTGGAA TGCGTTTTTC
1081   CTGGACAACC ATGACAATCC ACGTGCGGTG AGCCATTTCG GTGACGACCG TCCACAGTGG
1141   CGTGAACCGA GCGCGAAAGC CCTGGCAACC CTGACGCTGA CGCAGCGTGC GACCCCATTT
1201   ATCTACCAAG GCAGCGAGCT GGGTATGACG AATTATCCAT TTAAAGCGAT CGATGAGTTT
1261   GATGACATCG AAGTGAAAGG TTTTTGGCAT GATTACGTGG AGACGGGTAA GGTGAAAGCG
1321   GATGAATTCC TGCAGAATGT GCGCCTGACC AGCCGTGATA ATAGCCGTAC CCCGTTTCAG
1381   TGGGATGGCA GCAAGAATGC GGGCTTTACG AGCGGTAAGC CGTGGTTCAA GGTTAATCCG
1441   AATTACCAAG AAATCAACGC CGTTAGCCAG GTTACGCAAC CAGACAGCGT GTTCAATTAC
1501   TATCGCCAGC TGATTAAGAT CCGCCATGAT ATTCCGGCAC TGACCTATGG TACCTATACC
1561   GACCTGGATC CGGCCAACGA TAGCGTTTAT GCGTACACCC GTAGCCTGGG TGCGGAAAAG
1621   TACCTGGTGG TTGTTAATTT CAAGGAGCAG ATGATGCGTT ATAAACTGCC GGACAATCTG
1681   AGCATTGAGA AAGTGATTAT TGACAGCAAT AGCAAAAACG TGGTGAAAAA GAATGATAGC
1741   CTGCTGGAGC TGAAACCGTG GCAAAGCGGT GTTTACAAGC TGAATCAGTA A
```

Amino acid sequence of OmpA/palI

```
1      MPRQGLKTAL AIFLTTSLCI SCQQAFGTQQ PLLNEKSIEQ SKTIPKWWKE AVFYQVYPRS
61     FKDTNGDGIG DINGIIEKLD YLKALGIDAI WINPHYDSPN TDNGYDIRDY RKIMKEYGTM
121    EDFDRLISEM KKRNMRLMID VVINHTSDQN EWFVKSKSSK DNPYRGYYFW KDAKEGQAPN
181    NYPSFFGGSA WQKDEKTNQY YLHYFAKQQP DLNWDNPKVR QDLYTMLRFW LDKGVSGLRF
241    DTVATYSKIP DFPNLTQQQL KNFAAEYTKG PNIHRYVNEM NKEVLSHYDI ATAGEIFGVP
301    LDQSIKFFDR RRDELNIAFT FDLIRLDRDS DQRWRRKDWK LSQFRQIIDN VDRTAGEYGW
361    NAFFLDNHDN PRAVSHFGDD RPQWREPSAK ALATLTLTQR ATPFIYQGSE LGMTNYPFKA
421    IDEFDDIEVK GFWHDYVETG KVKADEFLQN VRLTSRDNSR TPFQWDGSKN AGFTSGKPWF
481    KVNPNYQEIN AVSQVTQPDS VFNYYRQLIK IRHDIPALTY GTYTDLDPAN DSVYAYTRSL
541    GAEKYLVVVN FKEQMMRYKL PDNLSIEKVI IDSNSKNVVK KNDSLLELKP WQSGVYKLNQ
```
