# Supplementary material for: Enhancing the Thermostability of Serratia plymuthica Sucrose Isomerase Using B-Factor-Directed Mutagenesis
Source: PLoS One. 2016 Feb 17;11(2):e0149208. doi: 10.1371/journal.pone.0149208 (PMC4757035; doi:10.1371/journal.pone.0149208)
Supplement: S3 File — The crucial conservation residues (Asp241, Glu295, and Asp369, His145 and His368) are indicated by hollow square. The unique short motif “RLDRD” of isomerization region in sucrose isomerase are indicated by red hollow rectangular. (PDF) [file pone.0149208.s003.pdf]

```
S.plymuthica AS9              1 MPRQGLKTALAIFLTTSLCISCQQAFGTQQPLLNEKSIEQSKTIPKWWKEAVFYQVYPRS
P.rubrum CBS574.77            1 MPRQGLKTALAIFLTTSLCISCQQAFGTQQPLLNEKSIEQSKTIPKWWKEAVFYQVYPRS
Enterobacter sp. FMB-1        1 MSFFKKLTGVAVTFSSLFMTLAPAAYSAETSVTQSIQTQKESTLPAWWKEAVFYQIYPRS
K.pneumoniae NK33-98-8        1 MSFVTLRTGVAVALSSLIISLACPAVSAAPSLNQDIHVQKESEYPAWWKEAVFYQIYPRS
S.plymuthica ATCC15928        1 MPRQGLKTALAIFLTTSLSVSCQQALGTQQPLLNEKSIEQSKTIPKWWKEAVFYQVYPRS
E.rhapontici NX5              1 MDSQGLKTAVAIFLATTFSATSYQACSAGPDTAPSLTVQQSNALPTWWKQAVFYQVYPRS
Klebsiella sp. LX3            1 MSFVTLRTGVAVALSSLIISLACPAVSAAPSLNQDIHVQKESEYPAWWKEAVFYQIYPRS

S.plymuthica AS9             61 FKDTNGDGIGDINGIIEKLDYLKALGIDAIWINPHYDSPNTDNGYDIRDYRKIMKEYGTM
P.rubrum CBS574.77           61 FKDTNGDGIGDINGIIEKLDYLKALGIDAIWINPHYDSPNTDNGYDIRDYRKIMKEYGTM
Enterobacter sp. FMB-1       61 FKDTNGDGIGDIRGIIEKLDYLKSLGIDAIWINPHYDSPNTDNGYDIRDYEKIMQEYGTM
K .pneumoniae NK33-98-8      61 FKDTNDDGIGDIRGIIEKLDYLKSLGIDAIWINPHYDSPNTDNGYDISNYRQIMKEYGTM
S.plymuthica ATCC15928       61 FKDTNGDGIGDIKGIIEKLDYLKALGIDAIWINPHYDSPNTDNGYDIRDYRKIMKEYGTM
E.rhapontici NX5             61 FKDTNGDGIGDLNGIIENLDYLKKLGIDAIWINPHYDSPNTDNGYDIRDYRKIMKEYGTM
Klebsiella sp. LX3           61 FKDTNDDGIGDIRGIIEKLDYLKSLGIDAIWINPHYDSPNTDNGYDISNYRQIMKEYGTM

S.plymuthica AS9            121 EDFDRLISEMKKRNMRLMIDVVINHTSDQNEWFVKSKSSKDNPYRGYYFWKDAKEGQAPN
P.rubrum CBS574.77          121 EDFDRLISEMKKRNMRLMIDVVINHTSDQNEWFVKSKSSKDNPYRGYYFWKDAKEGQAPN
Enterobacter sp. FMB-1      121 EDFDTLVSEMKKRNMRLMIDVVINHTSDQHPWFIQSKSSKENPYREYYFWRDGKDNQPPN
K .pneumoniae NK33-98-8     121 EDFDNLVAEMKKRNMRLMIDVVINHTSDQHPWFIQSKSDKNSPYRNYYFWRDGKDNQPPN
S.plymuthica ATCC15928      121 EDFDRLISEMKKRNMRLMIDVVINHTSDQNEWFVKSKSSKDNPYRGYYFWKDAKEGQAPN
E.rhapontici NX5            121 EDFDRLISEMKKRNMRLMIDIVINHTSDQHAWFVQSKSGKNNPYRDYYFWRDGKDGHAPN
Klebsiella sp. LX3          121 EDFDSLVAEMKKRNMRLMIDVVINHTSDQHPWFIQSKSDKNNPYRDYYFWRDGKDNQPPN

S.plymuthica AS9            181 NYPSFFGGSAWQKDEKTNQYYLHYFAKQQPDLNWDNPKVRQDLYTMLRFWLDKGVSGLRF
P.rubrum CBS574.77          181 NYPSFFGGSAWQKDEKTNQYYLHYFAKQQPDLNWDNPKVRQDLYAMLRFWLDKGVSGLRF
Enterobacter sp. FMB-1      181 NYPSFFGGSAWQKDDKTGQYYLHYFARQQPDLNWDNPKVRGDLYAMLRFWLDKGVSGMRF
K .pneumoniae NK33-98-8     181 NYPSFFGGSAWQKDVKSGQYYLHYFARQQPDLNWDNPKVREDLYAMLRFWLDKGVSGMRF
S.plymuthica ATCC15928      181 NYPSFFGGSAWQKDEKTNQYYLHYFAKQQPDLNWDNPKVRQDLYAMLRFWLDKGVSGLRF
E.rhapontici NX5            181 NYPSFFGGSAWEKDDKSGQYYLHYFAKQQPDLNWDNPKVRQDLYDMLRFWLDKGVSGLRF
Klebsiella sp. LX3          181 NYPSFFGGSAWQKDAKSGQYYLHYFARQQPDLNWDNPKVREDLYAMLRFWLDKGVSGMRF

S.plymuthica AS9            241 DTVATYSKIPDFPNLTQQQLKNFAAE---YTKGPNIHRYVNEMNKEVLSHYDIATAGEIF
P.rubrum CBS574.77          241 DTVATYSKIPDFPNLTQQQLKNFAAE---YTKGPNIHRYVNEMNKEVLSHYDIATAGEIF
Enterobacter sp. FMB-1      241 DTVATYSKIPGFPDLTPEQQKNFAEQ---YTTGPNIHRYLQEMKQEVLSRYDVVTAGEIF
K .pneumoniae NK33-98-8     241 DTVATYSKIPGFPNLTPEQQKNFAEQ---YTMGPNIHRYIQEMNREVLSRYDVATAGEIF
S.plymuthica ATCC15928      241 DTVATYSKIPDFPNLTQQQLKNFAAE---YTKGPNIHRYVNEMNREVLSHYDIATAGEIF
E.rhapontici NX5            241 DTVATYSKIPNFPDLSQQQLKNFAEE---YTKGPKIHDYVNEMNREVLSHYDIATAGEIF
Klebsiella sp. LX3          241 DTVATYSKIPGFPNLTPEQQKNFAEQ---YTMGPNIHRYIQEMNRKVLSRYDVATAGEIF

S.plymuthica AS9            298 GVPLDQSIKFFDRRRDELNIAFTFDLIRLDRDSDQRWRRKDWKLSQFRQIIDNVDRTAGE
P.rubrum CBS574.77          298 GVPLDQSIKFFDRRRDELNIAFTFDLIRLDRDSDQRWRRKDWKLSQFRQIIDNVDRTAGE
Enterobacter sp. FMB-1      298 GVPLERSSDFFDRRRNELDMSFMFDLIRLDRDSNERWRHKKWTLSQFRQIINKMDSNAGE
K .pneumoniae NK33-98-8     298 GVPLDRSSQFFDRRRHELNMAFMFDLIRLDRDSNERWRHKSWSLSQFRQIISKMDVTVGK
S.plymuthica ATCC15928      298 GVPLDQSIKFFDRRRDELNIAFTFDLIRLDRDSDQRWRRKEWKLSQFRQVIDNVDRTAGE
E.rhapontici NX5            298 GVPLDKSIKFFDRRRNELNIAFTFDLIRLDRDADERWRRKDWTLSQFRKIVDKVDQTAGE
Klebsiella sp. LX3          298 GVPLDRSSQFFDRRRHELNMAFMFDLIRLDRDSNERWRHKSWSLSQFRQIISKMDVTVGK
```

S.plymuthica **AS9**	358	YGWNAFFLDNHDNPRAVSHFGDDRPQWREPSAKALATLTLTQRATPFIYQGSELGMTNYP
P.rubrum **CBS574.77**	358	YGWNAFFLDNHDNPRAVSHFGDDRPQWREPSAKALATLTLTQRATPFIYQGSELGMTNYP
Enterobacter sp. **FMB-1**	358	YGWNTFFLDNHDNPRAVSHFGDDSPQWIEPSAKALATIILTQRATPFIFQGSELGMTNYP
K .pneumoniae **NK33-98-8**	358	YGWNTFFLDNHDNPRAVSHFGDDRPQWREASAKALATITLTQRATPFIYQGSELGMTNYP
S.plymuthica **ATCC15928**	358	YGWNAFFLDNHDNPRAVSHFGDDRPQWREPSAKALATLTLTQRATPFIYQGSELGMTNYP
E.rhapontici **NX5**	358	YGWNAFFLDNHDNPRAVSHFGDDRPQWREHAAKALATLTLTQRATPFIYQGSELGMTNYP
Klebsiella sp. **LX3**	358	YGWNTFFLDNHDNPRAVSHFGDDRPQWREASAKALATITLTQRATPFIYQGSELGMTNYP

S.plymuthica **AS9**	418	FKAIDEFDDI[illegible]KGFWHDYVETGKVKADEFLQNVRLTSRDNSRTPFQWDGSKNAGFTSGK
P.rubrum **CBS574.77**	418	FKAIDEFDDIEVKGFWHDYVETGKVKADEFLQNVRLTSRDNSRTPFQWDGSKNAGFTSGK
Enterobacter sp. **FMB-1**	418	FKKLNEFDDIEVKGFWQDYVQTGKVSAEEFIDNVRLTSRDNSRTPFQWNDRKNAGFTSGK
K .pneumoniae **NK33-98-8**	418	FRQLNEFDDIEVKGFWQDYVQSGKVTATEFLDNVRLTSRDNSRTPFQWNDTLNAGFTRGK
S.plymuthica **ATCC15928**	418	FKAIDEFDDIEVKGFWHDYVETGKVKADEFLQNVRLTSRDNSRTPFQWDTSKNAGFTSGK
E.rhapontici **NX5**	418	FKKIDDFDDVEVKGFWQDYVETGKVKAEEFLQNVRQTSRDNSRTPFQWDASKNAGFTSGT
Klebsiella sp. **LX3**	418	FRQLNEFDDIEVKGFWQDYVQSGKVTATEFLDNVRLTSRDNSRTPFQWNDTLNAGFTRGK

S.plymuthica **AS9**	478	PWFKVNPNYQEINAVSQVTQPDSVFNYYRQLIKIRHDIPALTYGTYTDLDPANDSVYAYT
P.rubrum **CBS574.77**	478	PWFKVNPNYQEINAVSQVTQPDSVFNYYRQLIKIRHDIPALTYGTYTDLDPANDSVYAYT
Enterobacter sp. **FMB-1**	478	PWFRINPNYVEINADKELIRNDSVLNYYKEMIKLRHKTPALIYGTYKDISPEDDSVYAYT
K .pneumoniae **NK33-98-8**	478	PWFHINPNYVEINAEREETREDSVLNYYKKMIQLRHHIPALVYGAYQDLNPQDNTVYAYT
S.plymuthica **ATCC15928**	478	PWFKVNPNYQEINAVSQVAQPDSVFNYYRQLIKIRHNIPALTYGTYTDLDPANDSVYAYT
E.rhapontici **NX5**	478	PWLKINPNYKEINSADQINNPNSVFNYYRKLINIRHDIPALTYGSYIDLDPDNNSVYAYT
Klebsiella sp. **LX3**	478	PWFHINPNYVEINAEREETREDSVLNYYKKMIQLRHHIPALVYGAYQDLNPQDNTVYAYT

S.plymuthica **AS9**	538	RSLGAEKYLVVVNFKEQMMRYKLPDNLSIE-KVIIDSNSKNVVKKNDSLLELKPWQSGVY
P.rubrum **CBS574.77**	538	RSLGAEKYLVVVNFKEQMMRYKLPDNLSIE-KVIIDSNSKNVVKKNDSLLELKPWQSGVY
Enterobacter sp. **FMB-1**	538	RTLGKERYLVVINFTEKTVRYPLPENNVIK-SILIEANQNKTAEKQSTVLTLSPWQAGVY
K .pneumoniae **NK33-98-8**	538	RTLGNERYLVVVNFEEYPVRYTLPANDAIE-EVVIDTQQQATAP-HSTSLSLSPWQAGVY
S.plymuthica **ATCC15928**	538	RSLGAEKYLVVVNFQEQVMRYKLPDNLSIE-KVIIESNSKNVVKKNDSLLELKPWQSGVY
E.rhapontici **NX5**	538	RTLGAEKYLVVINFKEEVMHYTLPGDLSIN-KVITENNSHTIVNKNDRQLRLEPWQSGIY
Klebsiella sp. **LX3**	538	RTLGNERYLVVVNFKEYPVRYTLPANDAIE-EVVIDTQQQAAAP-HSTSLSLSPWQAGVY

S.plymuthica **AS9**	597	KLNQ-
P.rubrum **CBS574.77**	597	KLNQ-
Enterobacter sp. **FMB-1**	597	ELQ--
K .pneumoniae **NK33-98-8**	596	KLR--
S.plymuthica **ATCC 15928**	597	KLNQ-
E.rhapontici **NX5**	597	KLNP-
Klebsiella sp. **LX3**	596	KLR--

Fig. S1. Amino acid sequence alignments of glycoside hydrolase family 13. The crucial conservation residues (Asp^{241}, Glu^{295}, and Asp^{369} ,His^{145} and His^{368}) are indicated by hollow square. The unique short motif “RLDRD” of isomerization region in sucrose isomerase are indicated by red hollow rectangular.
